# Differential Diagnosis of Autoimmune Encephalitis from Infectious Lymphocytic Encephalitis by Analysing the Lymphocyte Subsets of Cerebrospinal Fluid

**DOI:** 10.1155/2019/9684175

**Published:** 2019-12-03

**Authors:** Qiao-quan Zhang, Yan-fang Zhang, Nian Yu, Xing-jian Lin, Qing Di

**Affiliations:** ^1^Department of Pathology, The Affiliated Nanjing Brain Hospital of Nanjing Medical University, 210029 Nanjing, China; ^2^Department of Neurology, The Affiliated Nanjing Brain Hospital of Nanjing Medical University, 210029 Nanjing, China

## Abstract

This study is aimed at investigating the lymphocyte subsets of cerebrospinal fluid (CSF) to provide possible differential diagnostic values and better understand the pathophysiological mechanism underlying autoimmune encephalitis (AE) and infectious lymphocytic encephalitis. A series of CD markers, including CD3/4/8/20 representing different types and developmental stages of lymphocytes, were used to count the corresponding subpopulations of CSF from clinical and laboratory confirmed cases of anti-N-methyl-D-aspartate receptor AE (NMDAR-AE), herpes simplex virus encephalitis (HSVE), and tuberculous meningitis (TBM). The percentages of lymphocytes observed and the CD4 : CD8 ratios were compared between the three groups. There were no significant differences of the percentage of total lymphocytes, CD3 cells, and CD4 cells of CSF among each group. However, there were strongly statistical differences of the CD4 : CD8 ratio in CSF of each group with 0.6 : 1 in NMDAR-AE, 0.9 : 1 in HSVE, and 3.2 : 1 in TBM. The percentage of CD20 B lymphocytes in NMDAR-AE was statistically higher than that of other groups. The distinct percentages of lymphocyte subpopulations of CSF appeared to be characteristic and could potentially serve as diagnostic indicators. Further verification and research will be necessary to clarify the significance and nature of CD4 : CD8 ratios and B lymphocytes in CSF between AE and the infectious lymphocytic encephalitis.

## 1. Introduction

Autoimmune encephalitis (AE) is a group of newly recognized encephalitis syndromes associated with the autoantibodies to the antigen of neurons [1]. Among them, anti-N-methyl-D-aspartate receptor AE (NMDAR-AE) is known as the most common type usually affecting young females, but it also not rarely involved the children and elders [2]. A confirmatory diagnosis of AE relies on the detection of autoantibodies; however, the antibody tests can be negative in the early onset stage of AE [3]. Additionally, the pathophysiological mechanisms of NMDAR-AE are still incompletely understood [4]. So before the antibody is successfully detected, it is easily to be misdiagnosed as other diseases with the similar clinical manifestations or cerebrospinal fluid (CSF) characteristics, such as herpes simplex virus encephalitis (HSVE), tuberculous meningitis (TBM), syphilis meningitis (SM), and Creutzfeldt-Jakob disease occasionally. However, it had been evidenced that early diagnosis and treatment will predict a better prognosis for this treatable serious disorder of NMDAR-AE [5]. Additionally, it is unlikely to have relevant specific brain imaging abnormalities on the initial presentation or follow-up period of NMDAR-AE [6]. Therefore, it is necessary to find newly differential diagnostic methods on these disorders.

CSF examination is still an exclusive golden diagnostic method in patients with the CSN infectious diseases or AE [7]. But they usually manifest similar CSF findings with lymphocytic pleocytosis or an elevated protein in CSF. We have reported there were strong differences of CD4 and CD45RO T cells of CSF in SM and TBM patients; likely, there may be various pathways of immune dysregulation and an influx of activated lymphocytes into the CNS, resulting in corresponding lymphocytic encephalitis [8]. So far, there is no study reported to illustrate the differential subtypes of CSF lymphocytes among AE and the infectious lymphocytic encephalitis patients.

In the current study, the immunocytochemistry (ICC) method of CSF was extended to investigate the different percentages of lymphocyte subpopulations and CD4 : CD8 ratios in the CSF of patients with NMDAR-AE, HSVE, and TBM for new early diagnostic insights. Second, this study would also provide the explanations for the potential mechanisms of NMDAR-AE and the association with other infectious encephalitis from this regard.

## 2. Materials and Methods

### 2.1. Patients and Database

A total of 66 patients including 21 NMDAR-AE cases, 23 HSEV cases, and 22 TBM cases, were enrolled in this study. The basic clinical information is shown in Table 1. All the patients were admitted and diagnosed at our hospital during the three-year period between January 2016 and March 2019. This study has been ethically approved by the Affiliated Nanjing Brain Hospital of Nanjing Medical University.

The criteria for AE were based on a Lancet paper of “A Clinical Approach to Diagnosis of Autoimmune Encephalitis” [9] and “Chinese Expert Consensus for the Diagnosis and Treatment of Autoimmune Encephalitis” (Chinese Medical Association of Neurology, DOI: 10.3760/cma.j.issn.1006-7876.2017.02.004). The initial criteria to be met for the consideration of possible AE include are as follows: (1) a compatible clinical syndrome of subacute/rapidly progressive memory loss, psychiatric symptoms, or consciousness disorders; (2) one or more of (A) focal CNS findings, (B) new seizures, (C) speech disturbance, (D) severe dyskinesia, and (E) autonomic dysfunction; (3) electroencephalogram abnormalities; and (4) pathologic neuroimaging findings of MRI. Apart from the clinical information, the final definite diagnosis was made based on antibody testing results. Anti-NMDAR was tested in both the CSF and serum using an indirect immunofluorescence testing (IIFT) method. The postfixed sagittal mouse brain sections and IIFT detection kit in the form of biochips were commercially available from the company of Euroimmun (Lübeck, Germany). But at least, the CSF results of anti-NMDAR positive were the reference indicator.

The criteria for HSV was as follows [10]: (1) there are typical clinical features of viral infection, such as fever, headache, vomiting, seizures, and mental disorders; (2) the definitive etiological diagnosis of HSVE was based on anyone of the positive results being obtained in the following four laboratory tests: the nested polymerase chain reaction, chemiluminescence assay, specific intrathecal HSV antibody synthesis, or next-generation sequencing. The inclusion criteria of TBM have been described detailed in our previous study [8]. The capacity and security of pathogens testing in our hospital were authorised by the Jiangsu Province's Centre Disease Control and Prevention.

The excluded criteria were as follows: (1) the high proportions of nonviable cells or insufficient cell numbers are to be evaluated; (2) anti-NMDAR antibody was solely positive in CSF or blood, but without related clinical encephalitis evidence; and (3) when the first CSF was collected of the patient admitted, it was over than two weeks after disease onset.

### 2.2. Immunocytochemistry and Semiquantitative Analysis

#### 2.2.1. Inspection Methods

CSF samples were collected on the first day of admission without any special or nonspecial treatment as a natural history of the disease, including anti-virus, anti-TB, or any types of steroids. CSF evaluating team was blinded to the diagnosis. The slides of CSF were prepared as follows: (i) inhaling 200 microliters of CSF into a cell centrifuge (Statspin Cytofuge 12 or Cytospin 3) at a low speed of 1000 rpm/3 min (centrifugal radius: 13 cm); (ii) drying the slides at room temperature (RT); (iii) fixing them with cold acetone for 3-5 min at 4°C in the refrigerator; and (iv) setting aside to prepare for use. The total CSF leukocyte counts and the percentage of lymphocytes in the CSF were observed in each patient by using the Wright–Giemsa stain method with centrifuged sediment of the CSF as described previously.

#### 2.2.2. Immunocytochemical Staining Methods

The detail protocols were as follows: staining was performed using the SP kit according to the manufacturer's instructions. After 10 min blocked with 5% normal goat serum at RT, the prepared slide above was incubated with primary antibody. Mouse anti-CD3 monoclonal antibody (clone SP7, Catalogue No. RMA-0543) represents the mature T cell type. Mouse anti-human CD4 monoclonal antibody (clone 4B12, Catalogue No. MAB-0521) represents T helper cells or inducer T cells, as the functional cells for executing and regulating immune systems. Mouse anti-CD8 monoclonal antibody (clone SP16, Catalogue No. RMA-0514) is the marker of T cells responsible for directly killing infected and abnormal cells. Mouse anti-CD20 monoclonal antibody (clone L26, Catalogue No. MAB-0020) is the special marker of B cell. All the four kinds of primary antibodies were purchased from Maixin Bio Co., Ltd., Fuzhou, China.

The primary antibodies were at a dilution ratio of 1 : 100 overnight at 4°C. After washing, the slices were incubated with biotin-labelled second antibody, reagent-labelled goat anti-mouse, and anti-rabbit IgG polymer (as an enhanced polymer) (Catalogue No. Polink-2 DAB, ZSGB-BIO, Beijing, China) at 37°C for 30 min. And then, the immunoreactivity was tested with the avidin-biotin-peroxidase technique, using 3,30-diaminobenzidine as the chromogen.

The percentages of CD4, CD3, and CD8 T cells and CD20 B-positive cells were counted, respectively. The method has been described in our published work [8]. The slide was placed after ICC staining under JEK colour microscopic image analysis system, with three different smears of CSF sediments. Five different visions are randomly taken in each subset of CSF lymphocyte sample. The total number of lymphocytes was counted first, and then, the percentages of positive cells were analysed by manual counting to make qualitative comparison of the intensity of the lymph cell expression of the different groups.

### 2.3. Statistical Analysis

Measurement data were expressed as the mean ± SD. Qualitative data were described as percentages or rations. Differences among groups with continuous data were analysed by *one-way ANOVA*, and further multiple comparisons for post hoc tests used the *Least Significant Difference* (LSD) method. A *chi-square test* was used for analysing categorical variables. The *P* value reported was two-sided, and *P* < 0.05 was considered statistically significant. All analyses were performed using the SPSS software (version 13.0, SPSS Inc., Chicago, IL, USA).

## 3. Results and Discussion

### 3.1. Results

The distributions of age and gender were not statistically different among the three groups (Table 1). There were also no obvious differences of the time duration from the disease onset to the CSF collection and the oral body temperature at their admission. The mean total CSF leukocyte counts, the mean percentage of lymphocytes, and CD4 : CD8 ratios in the CSF of three groups are shown in Table 2. There were no significant differences of the percentage of total lymphocytes and CD3 cells of CSF among each group (*P* = 0.784 and *P* = 0.535, respectively); however, the total WBC count in the NMDAR-AE group is lower than that of the HSVE and TBM groups (both *P* = 0.000). This suggests that it may be difficult by using the traditional method of CSF cytology to different NMDAR-AE from the infectious lymphocytic encephalitis including HSVE and TBM if they are presented with the similar WBC count in CSF.

**Figure 1:**
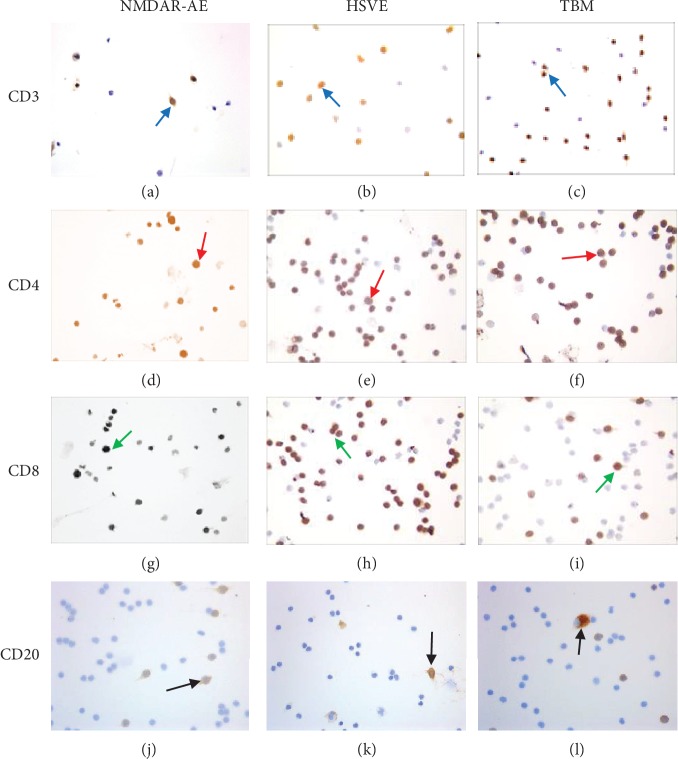
CD3, CD4, CD8, and CD20 in the CSF of patients with NMDAR-AE, HSVE, and TBM. The proportion of CSF CD20 cells was significantly higher in NMDAR-AE than that in other groups, while the proportion of CD3 cells was not obviously different among the three groups. Typical CD3 cells were marked in blue arrows in (a–c) for NMDAR-AE, HSVE, and TBM, respectively. Typical CD4 cells were marked in red arrows in (d–f) for NMDAR-AE, HSVE, and TBM, respectively. Typical CD8 cells were marked in green arrows in (g–i) for NMDAR-AE, HSVE, and TBM, respectively. Typical CD20 cells were marked in black arrows in (j–l) for NMDAR-AE, HSVE, and TBM, respectively.

The percentage of CSF CD4 T cell was lower in patients with NMDAR-AE than in patients in the HSVE group (*P* = 0.024), but with no significance within the TBM group (*P* = 0.220). The percentage of CSF CD8 T cell count was significantly higher in patients with NMDAR-AE (*P* = 0.0002) and HSVE (*P* = 0.000) than in patients with TBM. There were strongly statistical differences of the CD4 : CD8 ratio in CSF of each group with 0.6 : 1 in NMDAR-AE, 0.9 : 1 in HSVE, and 3.2 : 1 in TBM (all *P* = 0.000). The percentage of CD20 B lymphocytes in NMDAR-AE was higher than that of other groups. The proportion of CD20 B cells was not obviously different between the groups of TBM and HSVE (*P* = 0.650). These results suggested that the CD4:CD8 ratios and B lymphocytes in CSF may be useful for the differential diagnosis for the AE with infectious CNS disease (see Table 2, Figure 1).

## 4. Discussion

This study was to evaluate a method by CSF lymphocyte population analysis to assess the possible diagnostic differentiation of various neurological disorders. Based on the traditional CSF cytology method, we extended it to detect the lymphocyte subtypes using the ICC staining with monoclonal antibodies and further to compare the distribution CSF of lymphocyte subsets between autoimmune and infectious encephalitis. The current study showed that there were similar percentages of total and CD3 lymphocytes in CSF of the three groups observed. It is implied that it may be difficult to distinguish this lymphocytic encephalitis according to the traditional CSF cytology methods. Our results with the new method were clearly showing that there was a more predominance of B cells with the lowest CD4 : CD8 ratio in CSF of NMDAR-AE by compared with the other two kinds of infectious neurological diseases. At least, it will provide some clues for us when the antibodies have not been detected at the early stage of NMDAR-AE.

AE has been found closely associated with the special viral infection or the potential carcinoma [11, 12]. Recent studies show that some forms of AE can be developed from HSE [13, 14]. In the current results, there were many common characteristics of CSF between NMDAR-AE and HSEV patients, including the absolute CSF WBC count, the proportions of CD3 cells and CD8 cells, in spite of the different proportion of CD20 B cells.Previous studies also have showed that the patients with AE may involve B cell-mediated autoimmunity and the proportion of CD19 (+) B cells had been reported to be greater than 10% in CSF, which is significantly higher than that observed in noninflammatory neurological disorders [15]. Consistently, an immunohistochemical study showed prominent CD20 B cell cuffing was present around brain vessels accompanied by some plasma cells in a NMDAR-AE patient by performing a brain biopsy before immunomodulatory treatments [16]. The results above are in agreement with ours, with a higher proportion of CD20 B cells in the CSF of NMDAR-AE.

In our present study, we did not find significant differences of CD3 cell proportions of CSF in the three group patients including CNS infectious diseases with TBM, HSVE, and NMDAR-AE. Reportedly, there were higher proportions (51.4–77.0%) of CD3 cells of CSF in patients with the neurological disease (viral infection, neuroborreliosis and multiple sclerosis) compared with that in bacterial infection patients [17]. We speculated that this difference may be from the detected stages of the diseases or from the detected methods. This interesting phenomenon suggests us to perform further research on this topic using more samples and more precise methods.

The other useful information given by our present study is that the proliferation and differentiation of various lymphocytes may mediate the development of the CNS immune or infectious inflammatory disorders. T cells have different abilities to recognize antigen and are varied in terms of their function. CD4 cells represent a mature type of T cells [18]. CD8 cells, also called “killer” or cytotoxic T cells, perform the actual destruction of infected cells; CD4 cells play a regulatory role in immune response by assisting B cells to produce antibodies and assist killer T cells in their attack on foreign substances [19]. Consistent with our study, Adam et al. [20] had showed that AE being associated with CSF lymphocytic pleocytosis is usually milder than viral etiologies. Intratumoural CD3 and CD8 cell densities had also been proved to be prognostic factors for patients who have had surgically resected hepatocellular carcinoma [21]. According to the sequencing relationships between HSVE and NMDAR-AE, it is reasonable to speculate that there may be cellular immunity mediating inflammatory reaction dominated by the CD 4/8 T cells in the early detection of infection and then the cellular immunity above may be transferred to the humoral immunity dominated by the activation of B cells, which can lead to the production of antibodies associated with AE. Thus, further studies need to explore the subpopulations of CSF lymphocytes at different stages of AE and CNS infectious diseases.

## 5. Conclusions

In summary, the current study showed that there were different percentages of lymphocyte subpopulations and CD4 : CD8 ratios in the CSF of patients with NMDAR-AE, HSVE, and TBM, which may play an important potential role in diagnostic aspect. It may be helpful to use the immunocytochemistry method of CSF but it still need to be extensively verified in clinical practice with larger populations. This study also provides the clue of different types of immune response undergoing the pathophysiological mechanisms of NMDAR-AE and the association with other infectious encephalitis.

## Figures and Tables

**Table 1:** The basic clinical information of enrolled patients.

	NMDAR-AE (*n* = 21)	HSVE (*n* = 23)	TBM (*n* = 22)	Sig.
Sex (*n*/%)				Pearson's *χ*^2^ = 4.254, *P* = 0.119
Female	15 (71.43%)	14 (60.87%)	9 (40.91%)	
Male	6 (28.57%)	9 (39.13%)	13 (59.09%)	
Age yrs [mean ± SD (range)]	36.35 ± 19.89 (11-72)	31.02 ± 17.54 (10-68)	40.52 ± 18.36 (13-78)	*F* = 1.481, *P* = 0.235
Length [mean ± SD (range)]	10.45 ± 4.32 (3-18)	8.32 ± 5.68 (1-19)	9.56 ± 5.37 (2-17)	*F* = 0.981, *P* = 0.380
BT (*n*/%)				Pearson's *χ*^2^ = 0.533, *P* = 0.465
<37.3°C	2 (9.52%)	0 (0%)	1 (4.55%)	
37.3-38.9°C	16 (76.19%)	8 (34.78%)	16 (72.72%)	
>39.0°C	3 (14.29%)	15 (65.22%)	5 (22.73%)	

Note: “Length” means the time (days) from the disease onset to the CSF collection. “BT” means the oral body temperature at admission. Pearson's *χ*^2^ was obtained from chi-square test and *F* value was from one-way ANOVA.

**Table 2:** Comparison of T and B cell proportions in CSF of patients.

	NMDAR-AE	HSVE	TBM	*F*	*P*
WBC (/*μ*L) [mean ± SD (range)]	32 ± 21.27 (3-83)^#^^∗^	98 ± 46.32 (35-220)	155 ± 76.65 (65-278)	28.97	0.011
Lymph (%) [mean ± SD (range)]	46 ± 27.52 (30-99)	53 ± 38.24 (35-95)	48 ± 35.95 (12-90)	0.245	0.784
CD 3 cell (%) [mean ± SD (range)]	12 ± 9.47 (1-36)	14 ± 7.89 (2-27)	15 ± 9.56 (2-38)	0.632	0.535
CD4 cell (%) [mean ± SD (range)]	41 ± 25.24 (3-76)	59 ± 30.26 (12-89)	50.71 ± 20.50 (11-98)	2.66	0.078
CD8 cell (%) [mean ± SD (range)]	63 ± 31.59 (32-92)^∗^	66 ± 32.56 (21-98)	16.36 ± 7.21 (8-25)	18.95	0.001
CD4 : CD8 ratios [mean ± SD (range)]	0.6 : 1 (0.4-1.3)^∗^	0.9 : 1 (0.3-1.9)	3.2 : 1 (1.2-5.7)	59.96	0.002
CD20 B cell (%)	23 ± 12.59 (6.5-55)^#^^∗^	3 ± 2.87 (1-12)	2 ± 2.08 (1-11)	55.84	0.001

Note: WBC (/*μ*L) indicates the mean of the total CSF leukocyte counts; lymph (%) indicates the mean percentage of lymphocytes in the CSF. CD4 3 cell (%), CD4 T cell (%), CD8 T cell (%), and CD20 B cell (%) indicates each mean percentage in the CSF lymphocytes. CD4 : CD8 ratios mean the ratio between the percentages of CD4 and CD8 cells. ^#^*P* < 0.05 vs. the HSVE group; ^∗^*P* < 0.05 vs. the TBM group.

## Data Availability

The data used to support the findings of this study are included within the article.
